# Data sharing in neurodegenerative disease research: challenges and learnings from the innovative medicines initiative public-private partnership model

**DOI:** 10.3389/fneur.2023.1187095

**Published:** 2023-07-20

**Authors:** Angela Bradshaw, Nigel Hughes, David Vallez-Garcia, Davit Chokoshvili, Andrew Owens, Clint Hansen, Kirsten Emmert, Walter Maetzler, Lewis Killin, Rodrigo Barnes, Anthony J. Brookes, Pieter Jelle Visser, Martin Hofmann-Apitius, Carlos Diaz, Lennert Steukers

**Affiliations:** ^1^Alzheimer Europe, Luxembourg Ville, Luxembourg; ^2^Janssen Pharmaceutica NV, Beerse, Belgium; ^3^Department of Radiology and Nuclear Medicine, Amsterdam University Medical Center, Vrije Universiteit Amsterdam, Amsterdam, Netherlands; ^4^Bioinformatics Core, University of Luxembourg, Belvaux, Luxembourg; ^5^Institute of Psychiatry, Psychology and Neuroscience, King’s College London, London, United Kingdom; ^6^Department of Neurology, University Hospital Schleswig-Holstein, Campus Kiel and Kiel University, Kiel, Germany; ^7^Synapse Research Management Partners, Barcelona, Spain; ^8^Aridhia Informatics Ltd., Edinburgh, United Kingdom; ^9^Department of Genetics and Genome Biology, University of Leicester, Leicester, United Kingdom; ^10^Psychiatry and Neuropsychology, School for Mental Health and Neuroscience, University of Maastricht, Maastricht, Netherlands; ^11^Fraunhofer Institute for Algorithms and Scientific Computing, Sankt Augustin, Germany

**Keywords:** neurodegenerative disease, data sharing, innovative medicines initiative, GDPR, digital endpoint, real world data

## Abstract

Efficient data sharing is hampered by an array of organizational, ethical, behavioral, and technical challenges, slowing research progress and reducing the utility of data generated by clinical research studies on neurodegenerative diseases. There is a particular need to address differences between public and private sector environments for research and data sharing, which have varying standards, expectations, motivations, and interests. The Neuronet data sharing Working Group was set up to understand the existing barriers to data sharing in public-private partnership projects, and to provide guidance to overcome these barriers, by convening data sharing experts from diverse projects in the IMI neurodegeneration portfolio. In this policy and practice review, we outline the challenges and learnings of the WG, providing the neurodegeneration community with examples of good practices and recommendations on how to overcome obstacles to data sharing. These obstacles span organizational issues linked to the unique structure of cross-sectoral, collaborative research initiatives, to technical issues that affect the storage, structure and annotations of individual datasets. We also identify sociotechnical hurdles, such as academic recognition and reward systems that disincentivise data sharing, and legal challenges linked to heightened perceptions of data privacy risk, compounded by a lack of clear guidance on GDPR compliance mechanisms for public-private research. Focusing on real-world, neuroimaging and digital biomarker data, we highlight particular challenges and learnings for data sharing, such as data management planning, development of ethical codes of conduct, and harmonization of protocols and curation processes. Cross-cutting solutions and enablers include the principles of transparency, standardization and co-design – from open, accessible metadata catalogs that enhance findability of data, to measures that increase visibility and trust in data reuse.

## 1. Introduction

Data sharing is the process of making data available to people other than the data generators, collectors, custodians or stewards, forming a cornerstone of Open Science, wherein data is easily accessible, comprehensible, reproducible, replicable, and verifiable (1). Researchers and funding organizations are increasingly aware that data sharing is essential for effective and efficient biomedical research, and can also improve the accuracy and reproducibility of research, inform risk/benefit analyses of treatment options, strengthen collaborations, and enable large-scale analyses (2). Recognizing these practical and scientific benefits, journals in a variety of research fields, including medical science (3), have implemented data sharing policies, mandating data sharing statements and, in some cases, applying stringent requirements for data sharing.

However, these policy changes have not yet led to a substantial increase in data sharing from published research studies. For example, a cross-sectional analysis of 487 clinical trials published in *JAMA*, *Lancet*, and *New England Journal of Medicine*, reported that only 2 (0.6%) out of 334 articles agreed to data sharing, providing de-identified participant-level datasets or making them publicly available on journal websites. The same analysis also found that of the 89 articles stating they had provided individual participant data via a secure repository, only 17 articles had actually done so (4). Similarly, a 2021 study analyzing compliance of biomedical researchers with their Data Access Statements found that of 1792 manuscripts where datasets were “available upon reasonable request,” only 6.8% (123 manuscripts) provided the requested datasets upon request (5).

To further promote Open Science, the European Union (EU) has established minimal guidelines for data sharing in EU-funded projects (6). Under Article 29.2 of the Horizon 2020 model grant agreement, it was mandated to have unrestricted access to all peer-reviewed publications, including the right to download and print them. Moreover, a machine-readable electronic copy of the published version must be stored in a repository for scientific publications, together with bibliographic metadata providing the name of the action, project acronym and grant number (7). A similar provision to provide open access to peer-reviewed publications was also included in the European Innovative Medicines Initiative 2 joint undertaking (8). In Horizon Europe, the €95 billion Framework program for research and innovation that has succeeded Horizon 2020, the Open Science concept has been considerably expanded, imposing additional mandatory practices. These practices include an obligation to provide digital or physical access to the results needed to validate the conclusions of scientific publications, and an obligation to provide Open Access to research data under the principle “as open as possible, as closed as necessary” (9).

Alzheimer’s disease (AD) is the most common cause of cognitive impairment in individuals older than 65 years and is also one of the leading causes of death worldwide (10). According to estimates, neurodegenerative diseases such as AD are projected to create an economic burden of around €267 billion in Europe by 2030. The total cost of drug development for AD is estimated to be around $5.6 billion with an average duration of 13 years from preclinical studies to drug approval (11). Although advances in AD therapy have been achieved (e.g., FDA approval of aducanumab in 2021, as the first disease-modifying therapy for AD) (12), the failure rate in AD drug development remains very high (13, 14). Faced with such high human and economic costs, many public-private partnerships (PPP) have been established to improve the diagnosis, treatment and care of AD. As collaborative consortia which bring together key actors in the drug development process, PPPs are well-positioned to develop new therapies and lower the economic burden associated with devastating neurodegenerative diseases such as AD. From basic biomedical research and translational research to product registration and post-marketing surveillance, PPP aim to accelerate drug development by implementing non-linear, adaptive processes and strengthening collaborative approaches for the life-cycle management of therapies. Through a multidisciplinary and collaborative strategy in which stakeholders share knowledge, competencies, resources, and risks, PPPs have the potential to accelerate the translation of biological discoveries into clinical practice (12). PPP models can also identify new options to revisit discontinued products, call for funding for areas with unmet health needs, enhance knowledge of disease and promote learning from others, and sharing data (12). Since 2008, the Innovative Medicines Initiative (IMI), Europe’s largest public and private collaboration in the life sciences, has funded over twenty PPP on neurodegenerative diseases, accelerating research across a wide spectrum from preclinical science to applied clinical research.

In the field of neurodegeneration, the availability of data from small and large projects has resulted in unprecedented research and innovation (15) boosting the utility of data, accelerating research, and improving our understanding of disease causes, treatment, prevention, and care. Numerous initiatives for data sharing have been established, such as the Alzheimer’s Disease Neuroimaging Initiative (ADNI), Global Alzheimer’s Association Interrogation Network (GAAIN) and Alzheimer’s Disease Data Initiative (ADDI) in the United States, the Australian Imaging Biomarker & Lifestyle Flagship Study of Aging (AIBL) in Australia, the European Platform for Neurodegenerative Diseases (EPND) in Europe (16), the French National Alzheimer’s Information System, and SveDem-the Swedish Dementia Registry (17, 18). Funded through the IMI, projects such as the European Medical Information Framework (EMIF) and the European Prevention of Alzheimer’s Dementia [EPAD; (19)] have worked with research cohorts to undertake novel, large-scale research and develop systems and tools for data sharing (20). However, challenges in sharing data still remain.

Neuronet was a coordination and support action aimed at supporting and integrating projects in the IMI neurodegenerative disorders (ND) portfolio. Working on various themes and across different disease areas, twenty-four projects and 270 distinct organizations form the IMI neurodegeneration portfolio, including over 140 academic institutions, thirty-three companies that are members of the European Federation of Pharmaceutical Industries and Association (EFPIA), 55 SMEs (small and medium-sized enterprises) and 7 patient/carer organizations, among others (21). Neuronet aimed to support projects of the ND portfolio, to multiply its impact and visibility while enabling synergies and collaborations between partners in Europe, and around the world.

A Working Group (WG) on “data sharing and reuse” was established by Neuronet in 2019, bringing together experts from IMI ND projects. WG members were nominated by their respective projects (ADAPTED, AETIONOMY, AMYPAD, EMIF, EPAD, IMPRiND, PD-Mitoquant, RADAR-AD, RADAR-CNS) based on their expertise and experience in data sharing, with representatives from European academic institutions, industry, SMEs and patient organizations (further details on the WG composition and activities can be found on the Neuronet website). Experts contributed to discussions during quarterly online meetings, and also participated in a face-to-face workshop organized by Neuronet partners in early 2020, prior to the COVID pandemic. The WG aimed to share lessons learned, discuss common challenges and needs, and identify priorities and opportunities for synergy and collaboration across projects, with the expectation of having more consistent and informed decision making, improved reuse of results, improved networking across projects, greater exposure to expert knowledge, and more uniform application of standards. In this policy and practice review, we outline the challenges and learnings of the WG, providing the neurodegeneration community with examples of good practices and recommendations on how to overcome obstacles to data sharing.

## 2. Challenges and enablers for data sharing: insights from the Neuronet WG

Sharing data has the potential to improve public health in several ways, including facilitating research that provides a more thorough understanding of health issues, enabling the creation of innovative solutions, and ensuring that decisions are grounded on the best available evidence (22). PPP projects have great potential for data discovery and exchange to maximize innovation, but are subject to particular obstacles linked to their cross-sectoral scope and scale. These issues, which are influenced by different expectations and regulations at the funder, institution and state levels, were the subject of extensive discussions in the Neuronet WG on Data Sharing. Where relevant, discussions involved experts from other IMI projects outside the ND field (e.g., BigData@Heart, FAIRplus), who were invited to describe data sharing challenges they had encountered and resolved.

**Figure 1:**
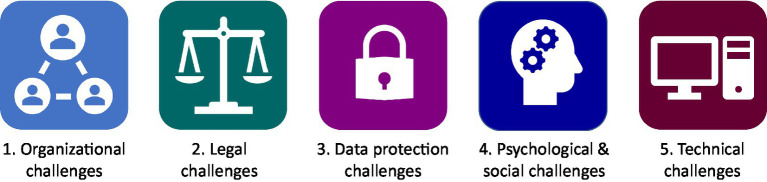
Challenges to data sharing in IMI neurodegeneration research projects. The Neuronet Working Group on data sharing identified five main categories of challenges that can impede data sharing (listed above), providing recommendations on how to address these challenges based on experiences of participation in IMI neurodegeneration projects (Boxes 1–5).

In this section, we outline the key learnings from these discussions, identifying key challenges, ways to address them, and providing examples of good practices from IMI PPP projects. Five main categories of challenges were identified by the WG, related to organizational and legal, data protection, psychological/social and technical issues. It should be noted that the challenges and good practices are primarily presented from a European perspective; for example, discussions on data protection are centered on the EU General Data Protection Regulation (GDPR), which regulates the processing of personal data from European citizens (Figure 1). Beyond the legal context, however, many data sharing challenges, barriers and enablers are shared between Europe and the rest of the world, expanding the relevance and utility of this review. Similarly, while the review draws primarily on experiences from sharing clinical data about human research participants and patients, there are overlaps in challenges experienced when sharing preclinical data.

### 2.1. Organizational challenges

From direct patient-clinician interactions to the research institutions or healthcare organizations involved, clinical studies in PPP projects on neurodegenerative disease typically have a complex hierarchy of relationships. These institutions or organizations may be part of regional consortia, provide data to a repository, or may be involved in data sharing networks. As a result, agreements on data sharing become multi-layered, multi-partner documents that are built on an initial agreement between patients and clinicians. Interactions between stakeholders at various levels of this hierarchy can therefore impact data sharing, influenced by sociotechnical factors such as trust. For example, a narrative review of empirical evidence addressing views and attitudes toward the use of health data for research reported that, despite being aware of the potential benefits of data sharing, participants were concerned about potential breaches of confidentiality and data abuses (23).

The organizational challenges linked to data sharing in clinical PPP studies exist at multiple levels and are influenced by questions surrounding rights to the data. For example, in clinical trials and cohort studies, participants have rights as data subjects, while also having a relationship with the clinical sites they visit, as well as the organizations with whom the data gets shared. In studies involving the use of real-world data (RWD), patients have rights as data subjects, maintaining interpersonal relationships with the clinicians involved in their care, and the hospitals or facilities where healthcare interventions take place. Consequently, there are particular challenges linked to the way individual studies are structured or governed, further complicated by the different objectives, interests and incentives for data sharing as viewed by the diverse range of institutions that participate in PPP consortia.

#### 2.1.1. Addressing organizational challenges

To address the organizational challenges outlined above, it is important to first have a good understanding of the placement of individual actors within the PPP or organizational structure, the laws, rules and regulations to which they are subject, and the aspects of data sharing each actor controls (Table 1). Organizational challenges may arise when the role of the different parties in data sharing agreements are unclear or not sufficiently defined. To address this issue, and under data protection legislation, the transactional roles of individual parties should be clearly defined. For example, data controllers must be identified by name in clinical studies, with principal investigators (PIs) or clinical research sponsors at a research institution often taking this role. Organizations or individuals with data processing roles should also be identified (e.g., legal entities providing technical services). Likewise, other roles that may be involved in data sharing, including data custodians (individuals who manage the data), data stewards (individuals who are responsible for the quality and correct usage of the data) and the data recipient (individuals or parties to whom the data is disclosed) should also be defined.

**Table 1:** Different actors in the organizational model.

	Legal basis	Data sharing degrees of freedom
Citizen	National and international law.	Can give consent to data sharing models, case by case. Can control downstream use of data (under GDPR).
Clinical Researcher	Staff contract, professional qualification.	Staff contract, professional qualification.
Medical Research Organization	Legal entity, subject to regulation in legal territory, e.g., as a charity or registered as a data controller.	High degree of freedom. Acts as data controller on receipt or creation of data. Can share data with researchers or subcontractors. Can take custody of 3rd party data on behalf of researchers. Can initiate and collaborate on projects with data sharing.
Pharmaceutical Company	Legal entity, subject to regulation in legal territory including company law.	High degree of freedom. Acts as data controller on receipt or creation of data. Can share data with internal researchers or subcontractors. Can initiate and collaborate on projects with data sharing.
Consortium	Partnership agreement.	Partnership agreement establishes a clear and usually constrained framework for data sharing inside and outside the protocol of a study.
Data sharing network	May be a legal entity (often not).	If legal entity, can contract data processors and facilitate and host data sharing agreements.

Organizational issues may also arise when individual parties are unable to act in the role required by legal frameworks that govern data sharing in PPP projects. To address these issues, as well as clearly defining which role each party plays, it is important to ensure each party has sufficient resources to fulfill that role. Organizations and individuals should invest sufficient time in training to effectively operate within this framework. The capability maturity model (23) is an organizational IT improvement strategy which could be applied to facilitate data sharing and collaboration. For example, it can be helpful to incorporate methods to obtain and record continuous feedback from individuals fulfilling different roles when sharing data, then use this feedback to adapt data flows, processes and infrastructures to facilitate data sharing. This can also identify process improvements and training needs to share data more effectively, providing paths for interactions and dialogs between organizational units and individuals to clarify priorities, requirements and limitations.

To further mitigate organizational issues in a sustainable way, researchers can also consider depositing de-identified data in a repository for long-term data preservation, creating a public record of the deposition, and formal metadata (e.g., digital object identifier (DOI) for citation) that can be more easily shared. This is still possible for datasets that require controlled access measures and can provide numerous advantages over managing data use agreements (DUAs) by email, with the platform handling aspects such as user registration, providing access to the DUA, enabling audit trails, etc. Not only does this remove some administrative burden for the researcher, but it also removes the reliance on the PI being available in perpetuity to deliver the data, and can improve record keeping and compliance.

**Box 1: Recommendations on addressing organisational challenges to data sharing.**

Organisational challenges

- Complex hierarchy of rela tionships between parties in PPPs, involving multiple actors across different sectors, who may have varying objectives, motivators, abilities and incentives to share data
- As a result, data sharing often involves multi-layered, multi-partner agreements that must satisfy intellectual property concerns in accordance with data protection regulations, reflecting this high degree of structural complexity.

Recommendations

- Transactional roles (e.g. principal investigators, data controllers, legal signatories) should be clearly defined and adequately resourced by PPPs, both financially and in terms of expertise
- Where suitable, applying a capability maturity model could help PPPs to define these actors and support their roles in data sharing processes, identifying and meeting training needs
- The use of existing Open Access infrastructures such as data catalogues, repositories or data sharing platforms can reduce some of the administrative burdens on individual researchers in PPPs, also supporting record-keeping, data governance and compliance.

### 2.2. Legal challenges

Discussions within the WG identified several legal issues that must be clarified when data is shared between beneficiaries, between IMI consortia, or with third parties. These include ownership of the data; access rights with conditions and usage limitations; possible embargo periods and associated time-limits for exercising access rights; how to provide access rights to affiliates, contractors or third parties; privacy restrictions; and ownership of, or access rights to results generated from shared data. To address these issues, many PPP projects establish additional legal agreements, some of which may be multi-party agreements that involve all consortium partners. Some contracts are mandatory due to the respective consortium agreements, and, in some cases, the process is streamlined by knowing who owns the data and who will be using it. However, these agreements are sometimes limited to specific purposes and are not wide-ranging to simplify and accelerate the process.

Beneficiaries of two IMI consortia can also enter into collaboration agreements to share specific data sets for particular purposes. In such scenarios, especially when all beneficiaries need to approve the collaboration agreement, the entire process becomes time-consuming and undermines timely collaboration. Data sharing agreements are also made between other beneficiaries, associated partners, linked partners, third parties, and other stakeholders. In a survey conducted by Neuronet to identify obstacles associated with project collaboration (24, 25), it was found that long delays involved in the preparation of agreeable terms and conditions for such collaboration documents and collection of signatures were the main issues.

#### 2.2.1. Addressing legal challenges

To share sensitive data sets with third parties, internal approval from business, intellectual property (IP), and regulatory groups involved in PPP projects should be obtained. This can help determine whether the data are proprietary or under license, and can identify potential use restrictions linked to research ethics (e.g., informed consent). Although challenges connected with research ethics/REC approvals were not addressed in discussions of the data sharing WG, the WG on Ethics and Patient Privacy identified the following enablers that may help address these challenges: (1) clearly identifying the roles and responsibilities of entities/individuals involved in clinical data collection, use, and storage (and providing concise explanations in consent forms), (2) adapting and aligning procedures for consent and management of data access requests across clinical sites through collaborative engagement with relevant site personnel; and (3) preparing multi-site study documentation with reference to prior REC approvals and involving REC experts where feasible, using accelerated processes (such as the Proportionate Review process in the UK) if available (26).

When sharing or reusing data, PPP consortia should discuss and evaluate the requirements for data privacy and data transparency (e.g., what data are sufficient to achieve the scientific objectives of research) and determine the appropriate level of data identifiability to be used (e.g., pseudonymised, anonymised, or synthetic), to support decision making that strikes a balance between data privacy and scientific value. It is also important to involve institutional legal teams from the early stages of the project, so that they know the context for any legal agreements needed; establishing policies and templates for data transfer and other data sharing agreements can help accelerate legal processes in a sustainable way. To this end, adequate resources need to be included in the projects, as the legal discussions can take months or years to solve issues.

**BOX 2: Recommendations on addressing legal challenges to data sharing.**

Legal challenges

- As cross-sectoral consortia involving multiple organizations of varying size, structure and complexity, PPPs raise particular issues around data ownership, access rights, usage limitations and privacy restrictions.
- Legal agreements in PPPs can be limited to specific purposes, with insufficient scope for application to the broad range of processes that can be involved in data sharing. Conversely, multi-layered, multi-partner agreements are often complex to negotiate and comply with, particularly when transactional roles of individual partners are not clearly defined.

Recommendations

- Where relevant, legal teams and/or signatories responsible for business, intellectual property and regulatory approvals should be identified, involved and informed from the early stages of PPP development.
- Establishing standard policies and template agreements for data sharing operations (e.g., data transfer agreements) in collaboration with all PPP partners can help contextualize and accelerate legal processes.

### 2.3. Data protection challenges

The Neuronet WG highlighted a number of challenges linked to the General Data Protection Regulation (GDPR; EU 2016/679), which regulates the sharing of personal data for health research in the EU, and came into force in May 2018. Under the GDPR, research participants in PPP clinical studies must be provided with information about how their personal data is collected, used, disclosed, transferred and retained. This information must be kept up-to-date, with material changes to the nature of data processing that impact on research participants’ legal rights and privacy risks to be communicated through appropriate privacy notifications. Deficiencies or unclear statements of consent forms used for research with human participants can result in publicly funded research data being unsuitable for sharing with other researchers (27). Apart from the right to be informed about how their data is used by researchers, participants also have the right to obtain a copy of their data and, under certain circumstances, can request for the transfer of the data in a portable format to an entity of their choice. Additionally, under the GDPR, research participants can influence whether, and/or to what extent, their existing (i.e., already collected) personal data can remain in use for future research projects. More specifically, participants may request their data to be deleted, if applicable, or alternatively, exercise their right to object to processing. These and other rights afforded to participants under the GDPR translate into corresponding obligations for medical researchers, thus increasing researchers’ overall legal compliance burden.

Although the GDPR was originally intended to simplify data sharing for societal benefit, certain provisions of the GDPR remain open for interpretation. Moreover, the GDPR does not provide specific guidance to clinical researchers (6, 28). Data sharing and reuse can fall under the provision of “further processing” under the GDPR, which imposes additional compliance requirements on researchers, with the situation further complicated by a lack of consensus over how articles and recitals relating to further processing should be interpreted (29). For example, although the GDPR deems further processing for scientific research purposes as a “compatible” form of data processing, currently there is no agreement on what this means in practical terms. In particular, a recent legal analysis by a group of privacy researchers has shown that “compatibility” of further processing should not be misconstrued to mean that further processing is necessarily permissible, or in GDPR terms, lawful (30).

Another major source of confusion within the clinical research community is the notion of consent as the legal basis for processing personal data under the GDPR. Consent, within the meaning of the GDPR, shares many similarities with the informed consent for participation in a medical study, a research ethics requirement. Nevertheless, the two types of consent are not the same, giving rise to somewhat counter-intuitive situations where although medical researchers routinely obtain informed consent from research participants, the participants’ personal data is processed under a GDPR legal basis other than consent (e.g., performance of a task in the public interest; Article 6 (1)(e) GDPR). Moreover, when consent is the GDPR legal basis for processing personal data in the context of medical research, it is unclear to what extent a valid consent can cover future, yet-to-be-specified research uses of the data. Recital 33 GDPR allows participants to consent “to certain areas of scientific research when in keeping with recognized ethical standards for scientific research,” thus seemingly obviating the need for study-specific consent. However, this interpretation has been expressly rejected by the Article 29 Working Party, the predecessor of the European Data Protection Board, the leading European authority tasked with interpreting provisions of the GDPR through its guidance documents (31). Several national data protection authorities, including, more recently, the Italian authority, have also reaffirmed that under the GDPR, a consent obtained at the time of data collection cannot be valid in relation to future unspecified research projects, thus necessitating a repeat consent (32). However, owing to the practical challenges associated with reconsenting research participants, this interpretation remains controversial within the medical research community, and has generated significant backlash in recent years (33, 34).

Finally, the GDPR, in particular Article 89(1) of the Regulation, broadly defines certain obligations, such as appropriate “technical and organizational measures” that must be complied with when processing personal data for scientific research purposes. However, the choice of, and compliance with technical and organizational measures to secure and pseudonymise data can be challenging for neurodegeneration PPPs, particularly when dealing with brain imaging and motion capture datasets where defacing and removal of other identifiers are required.

#### 2.3.1. Addressing data protection challenges

The lack of clarity around data protection policies and practices is an important barrier to data sharing among researchers, and was discussed at length by the Neuronet WG on data sharing. To address data protection challenges, it is crucial to confirm whether the consent forms permit sharing of study data with other researchers for secondary research purposes. Researchers should carefully consider potential uses of their research data when designing confidentiality agreements and consent forms, including long-term use, storage and sharing of the data (33). To support retrospective biomedical research using existing clinical datasets, the AD Data Initiative (ADDI) has created a decision tree to help researchers evaluate consent forms, to determine whether they permit data sharing (35). If this decision tree reveals that the consent form forgoes the desired data sharing or uses, potential alternatives can be investigated in collaboration with legal/administrative colleagues. An additional, useful resource is the Open Brain Consent Project, which was launched in 2014 to provide reference consent forms for data sharing, and tools to support pseudonymisation, has developed consent templates for researchers wishing to share brain imaging data, including a GDPR-compliant data consent form (36).

Researchers should also be aware that participant consent is not the only source of restrictions for data sharing. There can be additional constraints resulting from the needs of funding agencies (e.g., data cannot be shared for commercial reasons), various national laws (e.g., a separate ethics approval is necessary before sharing), or fundamental GDPR-related restrictions (e.g., data cannot be shared with parties relying on a particular legal basis to process data; or cannot be shared with parties in third countries). These constraints should be collaboratively identified and evaluated at the project outset, using and building on mechanisms such as Data Protection Impact Assessments (DPIA), and involving all key PPP project stakeholders, including data protection officers (DPOs) of the participating organizations.

**BOX 3: Recommendations on addressing data protection challenges to data sharing.**

Data protection challenges

- Data protection rights afforded to research participants under the EU’s General Data Protection Regulation (GDPR) can add a substantial burden of legal compliance for PPPs involving clinical research.
- A lack of specific guidance for clinical researchers, and the existence of Member State derogations in several important areas, has created a lack of clarity around consent parameters, lawful bases for data sharing, and technical and organizational measures to ensure patient privacy.

Recommendations

- Early evaluation of consent and clinical study documentation (from all sites, in the case of multi-site studies) by PPPs can help clarify the permitted use conditions for data and support the development of effective data sharing agreements.
- Researchers should consider the potential future uses of clinical datasets when designing confidentiality agreements, consent forms and other study documentation.
- From project outset, PPPs should analyze of all potential restrictions to data sharing (e.g., funding agencies specifying that data cannot be shared with commercial entities) in collaboration with project partners, building on mechanisms such as data protection impact assessments
- Early involvement of local data protection officers can help identify and overcome issues linked to local data governance policies in PPPs; similarly, federated governance structures can reduce administrative burdens that can arise with centralized data sharing platforms.

The IMI-funded Big Data@Heart project is creating a translational research platform on heart failure, acute coronary syndrome and atrial fibrillation, aiming to deliver scalable insights from RWD, clinical trials, cohort studies and patient registries. The BigData@Heart project combined data from a wide range of already-existing databases with advanced analytics to produce clinically relevant disease phenotypes (37). A number of learnings on how to address data protection and governance challenges were identified through this work. Networked or federated governance structures can reduce administrative burdens or delays that may arise with centralized governance structures. Excessive reliance on pre-specified local governance policies can hamper data sharing; early involvement of local data protection officers can add substantial value and efficiencies.

### 2.4. Psychological, social, and motivational challenges

Researchers have reported several psychological, social and motivational obstacles during data sharing. For example, in a survey conducted to understand the importance of data being discoverable, the authors reported an average rating of 7.3 on a scale of 1–10 (38). However, the concept of individual reputation and rewards can generate an exaggerated sentiment of ownership and competitive ‘loss’ associated with sharing and can create barriers, sometimes implemented as over-complicated access processes, or declining requests to share (39, 40). For example, a 2018 British Medical Journal (BMJ) study analyzing compliance of RCT investigators with BMJ and PLoS Medicine data sharing policies were only able to obtain data from 46% of 37 RCTs, with researchers either not responding to requests, or citing concerns relating to the financial cost and time required for the effort of data sharing (41).

In PPP projects, trust, trustworthiness, credibility, and reliance on systems already in place are further, crucial drivers and determinants of data sharing. This is especially true in the case of research consortia, where by definition of some level of sharing and collaboration is implicit in the work plan. For example, research participants must accept the risk of their data being compromised, and trust that clinical researchers will act honestly and to the best of their abilities, to maximize benefit for their patients. Similarly, researchers sharing data must trust that the data recipients will not misuse their data, and provide appropriate credit and acknowledgement for data generation. Group behavior is also an important factor; for example, the inexistence of a critical mass of peers sharing data can create an environment where there is a general reluctance to share as well, even without any objective obstacles. Reservations toward being the “first to share” are not uncommon.

#### 2.4.1. Addressing psychological/social challenges

Financial support for data sharing is not always provided by research funders, which also restrict financial support to the project duration. To address this issue, systems can be implemented to ensure that data sharing capabilities continue after the initial project, for example through continued funding from research funders, and/or sharing data through existing platforms such as the AD Workbench of ADDI or the Dementias Platform United Kingdom (DPUK) portal. This is an approach that has been successfully adopted by the IMI-EPAD project, which has provided open access to its longitudinal cohort study (LCS) datasets through the AD Workbench. These datasets include a wide range of cognitive, clinical, neuroimaging and biomarker variables from more than 2,000 participants in the LCS study.

Researchers could also take on the role of data stewards to receive credit for any reuse of their data, which could help incentivize continued involvement in data sharing and address motivational issues. In an ideal world, research systems should also ensure that researchers who share data are acknowledged and rewarded for doing so. For instance, a metric that measures the volume of data shared by researchers following findable, accessible, interoperable, and reusable (FAIR) principles (42) could be introduced, or funders could provide awards for researchers (as role models) for sharing their data. Ensuring appropriate recognition through the use of metrics and awards such as these could lead to “snowball” effects in terms of disposition to sharing, if they are used widely, consistently and in a highly-visible way.

COVID emphasized the importance, value, and feasibility of data sharing between research community stakeholders and organizations. Today, while there is a significantly higher level of preparedness and willingness to share data with researchers and policymakers to advance science, interpersonal relationships and parameters relating to trust still have the potential to impede data sharing. Trust and trustworthiness are therefore important considerations to address, for example by providing proof of the reliability of the research entity that is interested in the data, and by providing accessible, easy-to-understand information on how the processes, policies, procedures, and technologies work.

**BOX 4: Recommendations on addressing psychological and social challenges to data sharing.**

Psychological and social challenges

- The concept of individual reputation and reward, which is particularly prevalent in academic institutions, can generate an exaggerated sense of ownership and competitive “loss” when sharing data.
- Financial and technical costs of data sharing can act as additional disincentives, impacting motivation to share.

Recommendations

- Data DOIs, citations and metrics for data sharing and re-use can help incentivize data sharing, providing a mechanism for recognition and reward; similarly, researchers could act as data stewards to receive credit for any reuse of their data.
- PPPs could reduce the financial and technical costs associated with data sharing, and increase the visibility of their data sharing efforts, by using existing infrastructures for data sharing (e.g., the AD Workbench of the Alzheimer’s Disease Data Initiative).

### 2.5. Technical challenges

Although there has been rapid development in technologies to capture, manage, discover, standardize, visualize, analyze, and exploit data, technical challenges remain one of the key limiting factors impeding data sharing. A major problem is the fragmentation of the data landscape within PPP projects, which hinders interoperability and encourages new research projects to produce even more *de novo* innovations. The associated datasets are impacted by the numerous solutions that are not maintained or developed as a result. Every time a project tries to meet its unique needs while adhering to budget and time constraints, it must “reinvent the wheel,” which results in a sizable number of rudimentary solutions.

To maximize benefits from IMI-funded research projects, data should be available to external researchers, ideally in a format that is easily findable, accessible and reusable. These considerations extend to the metadata, which should help provide information as to the context of data collection, limitations of their applicability and interpretation notes, parameters that can hugely affect data reusability. However, curating data before analysis and sharing can require considerable effort, particularly when working with data from multi-site clinical studies or RWD, in different languages (both machine and human). For example, data harmonization involves ensuring the standardization of diverse datasets, removing errors and inconsistencies, and aligning on assumptions, syntactic and semantic interoperability. Several data harmonization methods can be used (each of them involves three operations: extract, transform and load), however, the processes are generally resource-intensive, particularly as the fidelity of the harmonization needs to be verified to enable further analyses. In addition, datasets need to be well-characterized (i.e., completeness, consistency and coverage) and the assumptions underlying the data need to be taken into account, ideally through collaborative processing with individuals who have domain expertise.

Other technical challenges arise for semi-structured and unstructured data, which require additional work, such as natural language processing. The choice of data sharing infrastructure also confers particular challenges; centralized infrastructures have advantages in terms of clarity of who is responsible for managing and organizing data, following in some cases an “honest broker” paradigm where trust and clear terms and conditions become key underpinning factors. However, they also have disadvantages in terms of implying the transfer of data to another location, which can be affected by problems of legal, ethical, governance and psychological nature and therefore requires an appropriate governance model. Federated infrastructures, where data is kept at source, with the data custodian as final arbiter on its use, have the advantage of more straightforward compliance with local legal and ethical rules and regulations. However, there are also disadvantages in terms of diluted responsibility, reliability and persistence of data, audit trail and also regarding the establishment and operation of unified access mechanisms for potential data users.

#### 2.5.1. Addressing technical challenges

Although there has been a significant push toward “open” solutions and “open” data in recent years, as well as the creation of numerous online repositories and catalogs, the adoption and reuse of tools and data heavily depend on appropriate provenance, context, and application domains. Support systems for data sharing need to provide details about the type of data being shared, where it came from, why it was collected, etc., all of which can significantly impact future analysis and interpretation. For this data, producers need to annotate, record, and provide as useful, effective, and actionable metadata as possible. Despite advancements in semantic web technologies, human input into the provision of such metadata remains crucial in many areas and requires enormous, frequently underappreciated efforts. To support these efforts, new ways to interact with data are being developed, such as machine learning tools to annotate metadata, as well as computational pipelines for improved visualization, analysis and comprehension of data. Here, the Neuronet WG identified several enablers for data sharing, which address the technical challenges outlined above.

##### 2.5.1.1. Addressing technical challenges: making data findable, accessible, interoperable, and reusable

Making data FAIR can supercharge how data are used. The IMI2 project FAIRplus was launched in 2019, to increase the FAIRification of valuable clinical datasets (43). Aiming to develop processes and guidelines on how to make data sets FAIRer, FAIRplus has created two tools for researchers to use: a FAIR Capability Maturity Model Integration (CMMI) and the FAIR cookbook. The FAIR cookbook which is hosted by ELIXIR (a European, distributed Research Infrastructure for life science data) collates protocols (termed “recipes”) for making data FAIR, targeted at researchers and data stewards. The FAIRplus CMMI incorporates these protocols, identifying different stages on the journey toward FAIRification of data and specifying protocols that can be used at different stages (from single-use datasets to standardized datasets and up to fully managed data assets, which are fully FAIR). To make data accessible in the long run, FAIRplus is applying its knowledge to the ELIXIR IMI data catalog at the University of Luxembourg (44), which will act as a searchable metadata repository of IMI data.

To support a metadata-driven catalog for FAIR data, it is crucial to identify all existing data that might have come from and are available from PPP projects and to share high-level information about such datasets. Numerous cataloging projects have been created as part of IMI neurodegeneration projects [e.g., EMIF Catalog, ROADMAP Data Cube, European Health Data & Evidence Network (EHDEN) data portal, EPND Catalog, AETIONOMY AData(Viewer)]. The ELIXIR-LU/eTRIKS Data Catalog, which is being created for major research initiatives like IMI and H2020 and is more expansive than the ND field, centralizes metadata of active and completed projects (45). Federated catalogs such as these allow users to discover the existence of data without accessing it, making them very helpful for facilitating requests for access to the desired data sets.

##### 2.5.1.2. Addressing technical challenges: harmonization

Data harmonization can be technically challenging, but is a strong enabler of data sharing, supporting FAIRification of data. The use of a common data model (CDM) to support harmonization and interoperability, for instance within a standardized, modular and extensible collection of data schemas, has gained considerable ground in recent times. Harmonization of vocabularies is integral to this process, especially within CDMs such as OMOP (Observational Medical Outcomes Partnership). The FDA’s Sentinel within a shared health data network (SHDN), the OMOP CDM within a federated or distributed network, or the Patient Centered Outcomes Research Institute (PCORI) CDM, are examples of such approaches, facilitating collaboration and harmonization of diverse data for analytics, in particular and for example, via a standardized analytics stack from OHDSI (Observational Health Data Sciences & Informatics) initiative, utilizing the OMOP CDM. OMOP is also at the centre of the EHDEN project, and, more recently, the DARWIN EU initiative of the EMA. Other established data standards to faciliate the sharing of structured data are also available, such as the CDISC SDTM and ADaM for clinical data, and SEND for preclinical data.

Within the IMI2 Big Data for Better Outcomes (BD4BO) initiative, individual projects, such as HARMONY (for hematological cancers), are mapping to the OMOP CDM, in this case via a pooled (centralized) SHDN, with Prostate Cancer Diagnosis and Treatment Enhancement Through the Power of Big Data in Europe (PIONEER) in prostate cancer working on mapping to the OMOP CDM via elements of a pooled SHDN and a federated SHDN, a hybrid model, or in the case of EHDEN a federated or distributed SHDN. The EHDEN project is unique in utilizing project-certified SMEs to undertake the extract, transform, load (ETL) with Data Partners, while working symbiotically with OHDSI on methodological, tools and use case development.

To support data harmonization, the EHDEN project identified a number of specific recommendations. Fundamentally, there needs to be a common understanding of the focus and standardized querying required for the common research proposed in a collaboration. In addition, the ETL process can be used to generate deeper insight into individual datasets while harmonizing, and is an excellent opportunity to have a feedback loop to the source for verification and improvements. During an ETL process, e.g., to the OMOP CDM, there should be a clear process for working between those knowledgeable of the source data and those responsible for the ETL, and clear verification and evaluation steps. Semi- or fully-automated steps and tools, with output reports during sequential steps and at the end of the ETL phase are important. Of note, with RWD on neurodegenerative diseases there will likely be a subset of variables harmonized, perhaps for specific queries, or for an ongoing program of research. Aligning on what will be harmonized is of paramount importance. Verification and evaluation of the fidelity between source data and harmonized data is good practice, in part with appropriate tools (integral to the OMOP CDM ETL process), but also in conducting validation studies, for instance by re-running protocols previously run in source data in the harmonized data. Utilizing standardized analytical tools assists with the preceding recommendation, and also assists with error detection with regards to whether an issue is with the source/harmonized data or the analysis, in particular with, e.g., higher dimensional data. Sharing the harmonization/ETL process, scripts, tools, and methods across the collaboration helps ensure complementarity of approach, even with a centralized ETL, while also educating relevant parties on the inherent steps and outputs. Harmonizing may be a one-off process, for instance with historical or static datasets, quite often with ND-RWD. With more dynamic datasets, the frequency of updates will need to be agreed upon, depending on the scope and scale of those datasets, and the ETL approach (e.g., to a CDM) could be semi or fully automated.

**BOX 5: Recommendations on addressing technical challenges to data sharing.**

Technical challenges

- The data sharing landscape within and between PPPs can be fragmented, with data stored in proprietary formats, in inaccessible locations, or with insufficient annotations, posing particular challenges for FAIRification of data.
- Processes such as data curation and harmonization, which facilitate data sharing, are resource-intensive; particular technical challenges may arise when sharing semi-structured and unstructured data, which may require, e.g., natural language processing.

Recommendations

- Mapping data to a widely-used common data model (e.g., OMOP) can support interoperability and facilitate data sharing in PPPs, while also enabling the use of standardized analytics across diverse datasets.
- Sharing harmonization processes, scripts and tools between PPP partners and with the wider research community can reduce the technical burden on individual researchers, build capacity, and break down silos.
- Open-source tools such as the FAIR cookbook can support FAIRification of datasets, providing protocols for assigning unique, persistent data identifiers, data transfer protocols, guidance on terminologies and ontologies for interoperability, and exemplars of data licences to permit data reuse.
- Using searchable, federated catalogs can be a resource-effective way to render PPP metadata findable, facilitating access requests and supporting data collaborations/sharing.

### 2.6. Learnings from data sharing in IMI neurodegeneration projects: real-world data, imaging datasets, and digital biomarkers

The previous section details the most prevalent challenges faced by neurodegenerative research PPPs, spanning organizational issues linked to the unique structure of these cross-sectoral, collaborative initiatives, to technical issues that affect the storage, structure and annotation of individual datasets. Equally, the learnings and recommendations that we outline are intended to be broadly applicable across different disease areas and research contexts. In this section, we focus on specific types of data that may be generated and shared by neurodegeneration PPPs: neuroimaging datasets, digital biomarker data, and clinical data that is routinely collected during healthcare delivery, also known as real-world data (RWD). Based on practical experiences from four IMI projects working with these datasets, we highlight particular challenges and learnings for sharing these datasets, identifying intersections with the five areas addressed in the previous section.

#### 2.6.1. Addressing sociotechnical concerns when working with RWD in neurodegenerative disorders: EMIF and EHDEN

The utilization of RWD for insight and evidence generation in a normative, observational setting outside of a clinical trial is not new, but has seen a remarkable expansion in recent years. The use of RWD is disease agnostic: the capture of clinical and associated data from diverse sources (phenotypic, genotypic or both) may bring new insights into our biology, right through to real-world outcomes of therapeutic interventions on disease progression. However, working with, sharing and reusing RWD comes with a number of sociotechnical challenges. It involves technical requirements to find, curate, and analyze data that is appropriate for the task. Likewise, it requires a sociological framework of governance, ethics, policy, and law to ensure that patients and citizens are adequately protected, and that data are available for research purposes.

The EMIF and EHDEN projects share the aim of scaling up the RWD ecosystem across Europe, to enhance the generation of reproducible and reliable evidence through large-scale, federated analyses of health data. EMIF, which was funded by the IMI between 2013 and 2018, developed a platform for electronic health records (EHR) and cohort-derived data, allowing users to find and explore these data sources. EMIF was divided into the platform development (EMIF-PLAT), metabolic focus (EMIF-MET) and in Alzheimer’s disease (EMIF-AD). EHDEN has leveraged elements of the EMIF catalog for its platform, and is also working to harmonize EHRs from millions of people to the OMOP CDM, in collaboration with institutions, data sources and data custodians across Europe. To date, EHDEN has created a network of over 187 data partners from twenty-nine different countries, which are mapping their data to the OMOP CDM in a federated network; in total, approximately 850 million EHRs are represented in this network, creating a hugely valuable resource for health data discovery, analysis and research.

With the experience gained from working on EMIF and EHDEN, the following recommendations have been put forward to address sociotechnical issues that may arise when working with RWD:

1. *Ethical guidance:* To enable relevant and compliant research within the framework of social norms, anyone working with RWD should acquire ethical guidance or employ an ethics advisory board. Any research utilizing RWD must strike a balance between risk and benefit for the individual, a cohort, and society as a whole.
2. *Compliance with regulations:* Legal advice must be obtained to ensure alignment with, for example, the GDPR, the Data Governance Act, derogated member state interpretations and regulations, and local institutional requirements.
3. *Transparency and federation:* The intended data use and research goal must be transparent to all parties, following local and regional permission requirements and governance standards, before the release of positive or negative findings. Federated systems have the distinct advantage of allowing for data custodians to apply their local governance frameworks, rules and regulations. Sharing only aggregated data through standardized tools minimizes privacy concerns. In addition, IT systems should be in place to avoid digital security threats and allow data to be accessed and shared safely.
4. *Public involvement:* Depending on the nature of the research, it may be possible to integrate meaningful patient and public involvement to provide guidance and direction on using RWD within the parameters of legitimate research and also account for social norms and diversity in its representation.
5. *Codes of conduct:* Instead of using several different techniques, overarching code(s) of conduct (e.g., the EMIF Code of Practice) can help ensure the consistent application of methods that adhere to ethical and data protection criteria across projects that use RWD. There are many guidelines available in Europe that promote the use of RWD in general and can be used to research practices concerning the nuances of working specifically with RWD.

The recommendations outlined illustrate how EMIF and EHDEN have met many of the *organizational, legal*, and *data protection* challenges detailed in the first half of this review, highlighting transparency and federation as an enabler for sharing RWD when supported by clear codes of conduct to support compliance with supranational, national and local regulations and laws.

#### 2.6.2. Sharing data from remote measurement technologies: remote assessment of disease and relapse – Alzheimer’s disease

Smart devices collect a wide variety of data from the wearer, such as daily activity patterns and levels, calories burned, sleep patterns and weight. Increased health awareness and greater use of smart devices have opened the door to using these RMT to evaluate patient outcomes, both to support the day-to-day management of health and as tools for clinical research. However, the collection, use and sharing of data collected via RMT entail particular technical, legal and ethical challenges.

RADAR-AD (Remote Assessment of Disease and Relapse - Alzheimer’s Disease) was launched by the IMI in January 2019 and will finish in June 2023. RADAR-AD aims to develop a digital platform that draws on a smartphone, wearable and home-based digital technologies to track subtle changes in the cognitive and functional abilities of people with AD. RADAR-AD is performing clinical studies that aim to assess different remote monitoring technologies and how the data that are generated using these technologies reflect the activities of daily living in people at different stages of AD. These data are being managed, stored and shared via the open-source RADAR-BASE platform, which was created during the RADAR-CNS project.

Data sharing and interoperability are firmly embedded in both RADAR projects. The framework supporting this data sharing (i.e., the type of data to be shared and access governing data sharing) was been established in line with IMI2 IP policy and considering the overall approach agreed upon in the other RADAR projects. EFPIA members and consortia partners are committed to sharing all data (clinical, biosensor etc.) available to, or generated by the RADAR program among all members of a RADAR topic, and across topics, as required. In addition to data, RADAR constituents also share domain practices and expertise developed concerning data management procedures, usability, regulatory and policy pathways etc. across the RADAR program and externally as required by IMI policy and procedures. It is expected that any system built within the RADAR program adheres to well-accepted data standards, where applicable, to ensure compatibility and interoperability with other systems both within the RADAR program and more widely. The developed solutions, irrespective of whether leveraging the foreseen facilitating common platform infrastructure or built independently from it, should, in any case, allow for cross-analysis, data stream sharing and aggregated visualization across all RADAR-AD solutions, as well as in combination with pre-existing solutions such as those being elaborated under RADAR-CNS.

With the experience gained from working on RADAR-AD, the following recommendations have been put forward:

1. *Data management planning:* Development of a data management plan before patient enrollment can help guide the management and sharing of patient and caregiver-generated RMT data according to FAIR principles, and should provide information about how study data will be handled during the project lifetime, the types of data that are being collected and shared, the standards and ethical policies for study data, and parameters for storage and retention of data during, and after the project.
2. *Data standards:* Any system developed for data curation, storage or management should adhere to widely known data standards, if applicable, to ensure compatibility and interoperability with other systems inside and beyond the RADAR initiative.
3. *Enabling collaboration:* Improving the process of acquiring access to datasets, which is usually time-consuming due to legal and ethical issues, can facilitate better research by promoting collaboration and multifaceted work.
4. *Sustainability and scalability:* The solutions developed should support cross-analysis, data stream sharing, and aggregate visualization across all RADAR-AD solutions and in combination with existing solutions such as those being elaborated under RADAR-CNS, regardless of whether they leverage the foreseen facilitating common platform infrastructure or are built independently of it.

The recommendations outlined illustrate how RADAR-AD has met *organizational, data protection*, and *technical* challenges relevant to sharing of data collected from remote measurement technologies including wearables and home-based digital technologies. Embedding interoperability and FAIRification through careful data management planning, application of well-established data standards, and use of modular, open-source platforms such as RADAR-BASE can support analysis across datasets, data sharing, and aggregate visualization.

#### 2.6.3. Working with imaging datasets: amyloid imaging to prevent Alzheimer’s disease

Alzheimer’s disease pathogenesis is characterized by the accumulation of amyloid-beta (A*β*) plaques, which is considered the first detectable change in the brain of a process that takes decades before the onset of the cognitive decline. In this context, amyloid positron emission tomography (PET) imaging has shown to be capable of capturing the continued accumulation of amyloid burden beyond the plateau observed in cerebrospinal fluid, and therefore it is an excellent tool that provides information about the topographical distribution and the burden of amyloid accumulation in the brain.

Although amyloid PET imaging holds great promise in a detailed characterization of the natural history of AD and its early stages, this technique must be accompanied by a well-phenotype description of the individuals. Despite the availability of longitudinal data sets on AD, such as ADNI, there was a need for large-scale (semi) quantitative amyloid PET data collected in the early population, where the pathological signal is often subtle. Therefore, the AMYPAD Prognostic and Natural History Study (PNHS) was established to build on existing cohorts, reducing the burden of *de novo* participants (46).

The AMYPAD PNHS data collection is a combination of prospective and historical data from twenty European sites in 8 different countries. These sites have provided information through eleven parent cohorts (PC) (47).

The “organizational” challenge was one of the first difficulties faced in the early stages of the project. AMYPAD PNHS was defined as an additional layer for existing PCs, providing financial support to perform an amyloid PET scan. As expected, this design was a source of organizational difficulties to define the “legal” framework governing the research data and ensuring that “data protection” aspects were well covered. A data transfer agreement template was used across PCs to facilitate and speed up the legal discussion. Additionally, regular updates and open discussions were maintained with all the PIs and members of the consortium on the different aspects of the project, to build trust in the project and overcome any psychological barriers. This communication channel was supported by allocating resources within the sponsor team to include the roles of project manager, research coordinator, and data manager. The support of this “sponsor team” was crucial to overcoming the challenges faced during the project, for example during the COVID period.

The participation of the different PI and their PCs facilitated the process of making the data available during the life of the AMYPAD project, as defined by the IMI grant. In contrast, challenges were more prominent to define the aspects surrounding data sharing after the IMI period, and most of the concerns presented above in this manuscript were manifested, such as data access request process, access rights or usage limitations.

To face this challenge, the AMYPAD PNHS dataset was defined with a sufficient degree of granularity to account for different research scenarios and the restrictions established by the PCs. Specifically:

1. *Data minimisation:* The variables included in the data set were grouped into concepts (i.e., common ideas or measurements) and domains (i.e., groups of concepts that share common characteristics). This allows the researcher to navigate the information available and enables access only to the subset of information needed to address the research question.
2. *Data protection:* The variables were further classified as *source* (i.e., original data shared by the PC), *raw* (i.e., minimally processed data, such as years of education, body measures or score in neuropsychological tests), *harmonized* (i.e., processed data harmonized across centers, such as x-scores or categories), and *derivative* (i.e., metrics obtained from neuroimaging processing methods). This division provided the project with three scenarios for data access requests: (1) *source* data will not be shared by AMYPAD PNHS, and the researcher needs to request access directly to the PC; (2) *raw* data will be shared only under direct approval by the PC; (3) sharing *harmonized* and *derived* data will require only the approval by an internal AMYPAD committee, while the PC will be kept informed.
3. *Data sharing platform*: To ensure the preservation of the data after the finalization of the IMI period, the AMYPAD PNHS established a 5-year partnership with the ADDI. Researchers interested in using the AMYPAD PNHS data will be able to request access to imaging and clinical data for scientific research and/or educational activities using the AD workbench platform.
4. *Use of standards*: Due to the variety of sources and data formats present across the PCs, the data curation process in PNHS has dealt with multiple challenges. Among those, the most notable was the use of different data models, measurements, and cognitive questionnaires by the PC. Therefore, it was decided to perform a comprehensive process of data curation based on the work of the Data Curation Network,^1^ which developed a standardized set of Check, Understand, Request, Augment, Transform, Evaluate, and Document (CURATED) steps. This integration process, and the strategies used for data transformation and harmonization, will be documented in a manuscript that will serve to understand the rationale followed during the study and, hopefully, will give guidance to future researchers that faced similar projects.
5. *Harmonized protocols:* The acquisition of amyloid PET data across different sites (e.g., a variety of PET and MRI scanners and acquisition protocols) presented a challenge to harmonizing the results obtained during image analysis. To tackle this, a specific Work Package was devoted to defining a protocol to harmonize the quantification of the amyloid PET imaging, a task performed in close collaboration with the EANM Research GmbH (EARL) initiative, from the European Association of Nuclear Medicine (EANM).
6. *Harmonized data:* finally, clinical research data will be shared using two different data models: first, using a flat-file model defined during the integration process in AMYPAD PNHS and, second, using the OMOP CDM that will allow the analysis of the data in combination with other databases that use the same common format. For neuroimaging data, the images will be shared using the directory structure, file naming, and metadata convention proposed by the Brain Imaging Data Structure [BIDS; (48)].

The recommendations outlined illustrate how AMYPAD has met *organizational, data protection*, and *technical* challenges that may arise when sharing and reusing brain imaging data, for example in defining legal frameworks, achieving GDPR compliance, and determining data access rights and processes. As highlighted in the previous sections on real-world data and data from remote measurement technologies, development of harmonized protocols, use of data standards, and defined data models can help address these issues; in addition, outlining potential scenarios for data access requests allowed AMYPAD to establish robust processes to enable data sharing.

## 3. Discussion and conclusion

Although the value of sharing data is widely acknowledged in the ND research community, multifaceted challenges remain, with public-private partnerships facing particular organizational, legal, data protection, social/psychological, and technical hurdles. Strategies to overcome specific hurdles may not improve data sharing if related barriers are not addressed comprehensively, or if the underlying systemic issues are not resolved. The goal of this policy and practice review was to provide a broad overview of common issues and dimensions related to data sharing and effective reuse, from the perspectives of experts working in IMI projects on neurodegenerative diseases. Our analysis highlighted a number of barriers inherent to large-scale, cross-sectoral and transnational research projects, starting with the complex hierarchy of relationships between partners, which impacts data sharing at several levels. With organizations that range in size from small groups of people to multi-national companies with thousands of employees spread across different divisions, there can be a lack of clarity and transparency in the roles and responsibilities of key actors in data sharing processes. As well as raising particular issues around data ownership, access rights, intellectual property and usage limitations, the involvement of both public and private partners means that multi-layered, multi-party agreements are often required, which are particularly complex to negotiate and comply with when the transactional roles of individual partners are not clearly defined.

Challenges caused by a lack of clarity on roles and responsibilities are further compounded by the lack of specific guidance on the governance of highly-sensitive clinical research data generated by PPPs. Regulations such as the GDPR are viewed by some as a double-edged sword, creating stringent rules for data protection, but not providing precise guidance on key requirements such as consent parameters and technical and organizational measures for pseudonymisation. This imposes a substantial burden of legal and ethical compliance on researchers and PPP partners, adding an extra layer of complexity that can hinder the establishment of essential contracts such as data transfer agreements. Indeed, some IMI projects reported negotiation periods lasting a year or more, with multiple rounds of review involving several legal teams. As a result, data governance - an essential requirement for effective data sharing - can become a highly-charged issue fraught with perceived risks, negatively impacting the motivation of researchers to share data.

Our review also identified systemic barriers to data sharing in PPP projects, which can create an unfavorable environment for fruitful collaboration and innovation. 141 of the 270 organizations partnering in IMI neurodegeneration projects are academic institutions. Academic metrics for impact, reputation and reward are primarily centered on the individual, measuring research parameters such as scientific publications and grant income. This can generate an exaggerated sense of data “ownership” and competitive loss when sharing data in and from PPPs, a sense that is further amplified by the legal and ethical burdens discussed in the previous paragraph. As a result, researchers understandably report that they are more prepared to trust existing collaborators, or high-profile researchers and institutions, which can create research silos that limit wider data sharing and collaboration. Indeed, silo-ing is a major issue at several levels: Neuronet WG members reported challenges due to organizational and collaborative silos, as described above, but also due to technical silos, where data discovery and sharing is restricted due to the use of proprietory formats and annotations, or inaccessible locations behind institutional firewalls. Curating data before analysis and sharing can require considerable effort, particularly when working with data from multi-site clinical studies, neuroimaging datasets, or RWD. Consequently, projects may resort to “in-house” data standards, processing pipelines and curation methodologies that may negatively impact semantic interoperability and harmonization, further limiting the potential for data sharing.

The Neuronet WG on data sharing was created to share lessons learned, discuss common challenges and needs, and identify priorities and opportunities for synergy and collaboration across projects. As such, we identified several enablers for data sharing in PPP projects, which can help overcome the challenges and barriers described above (also summarized in text Boxes 1–5).

Transparency was highlighted as an important facilitator at several levels. At the organizational level, transparent data governance processes with clear allocation of roles and responsibilities among PPP partners can accelerate data sharing, facilitating the establishment of agreements and contracts. In addition, using searchable, federated catalogs can be a resource-effective way to render PPP metadata findable, facilitating access requests and supporting data collaborations. Transparency should also extend to communicating about data sharing processes with key stakeholders, including research participants and the general public along with PPP partners and the wider neurodegeneration research community. As well as meeting ethical and legal requirements for informed consent and consent to data use, this can increase the visibility of, and public trust in, data sharing. Working Group members noted that this could also help bring about systemic changes in how data sharing is viewed, recognized and rewarded in academia. Increasing the visibility of data sharing, and emphasizing the moral imperative to share data from ND research studies, could lend further support to the adoption of metrics for data sharing. Metrics could include data access requests or publications that cite the use of shared data, facilitated by identifiers such as data DOIs (such as those assigned by Elsevier’s “Mendeley Data” platform) that allow data to be cited and shared in a visible way. Complemented by existing metrics such as publications, impact factors and grant income, adoption of these metrics by academic systems would boost collaboration and further enhance awareness and recognition of data sharing.

A second common theme when discussing facilitators for data sharing was standardization. The establishment and use of templates for cross-consortium agreements, where feasible, was identified as a way to accelerate legal and administrative processes for all involved parties, particularly when templates incorporate pre-existing clauses required by institutions or companies. Efficiencies could also be gained by harmonizing data and metadata, by mapping to widely-used common data models such as OMOP (as exemplified by the EHDEN project), preceded where necessary by comprehensive data curation using standardized steps (e.g., the CURATED approach used by AMYPAD). Integration of curation processes, and aligned strategies for data transformation and harmonization, were identified as important enablers of interoperability. Sharing of these processes, scripts and tools between PPP partners and other researchers can also help build capacity in the wider community, reducing the technical burden on individual researchers, breaking down silos, and reducing redundancy. For example, open-source tools such as the FAIR cookbook (developed by the FAIRplus project) can enable FAIRification of datasets, while avoiding “reinventing the wheel” for each successive PPP project. Similarly, using existing, federated platforms for sharing data (such as ADDI’s AD Workbench) can ease access to datasets and increase interoperability, while providing free computing power for analysis and re-use. However the principle of standardization should not be limited to legal agreements, technical tools and platforms. Experiences from IMI projects including EMIF and EHDEN emphasized the value of developing ethical guidelines and codes of conduct for PPPs, which help researchers to navigate some of the ethical complexities that may arise when sharing or reusing data.

The value of involving patients in all aspects of research - from development, to design and delivery – is now widely recognized (49, 50). While patients are usually not directly involved in data sharing, as the ultimate beneficiaries of research, and as data subjects, there is an ethical imperative to ensure patients’ needs and preferences are respected. Working Group members agreed that patient and public involvement (PPI) can provide valuable guidance and directions on sharing and re-using patient data in research. Involving patients in the design of protocols, agreements and processes can also increase public trust in data sharing. An equally important enabler for data sharing is the early consultation of key stakeholders in data sharing processes, such as data protection officers, legal signatories, database managers and clinical research coordinators. Involving these individuals from the PPP proposal stage onwards can help anticipate potential challenges, and identify ways to overcome them. For example, early involvement of local data protection officers can identify issues linked to local data governance policies, and consultations with clinical research coordinators can help clarify the perimitted use conditions for data.

Although our analysis has identified a number of practical enablers, a mindset shift in the research community is still required to advance data sharing more effectively. In particular, the community needs to reconsider who should be responsible for data management after the end of PPP projects. Technical, financial and administrative costs of data sharing can be prohibitive once project funding periods have ended. Could funding agencies therefore take on the role of data managers? At least at first glance, some advantages can be derived from this: state- or community-of-states-led initiatives are less subject to end-date risks. The interests are clearly on the side of the most frequent and effective use of the collected data (and less on the side of potentially existing self-interests of those who have collected the data), and the repository could thus also reach a critical size, which could lead to a self-perpetuating process concerning data collection and data analysis networks. Finally, there is growing awareness that federated networks can potentially bypass legal, organizational and sociotechnical issues linked to ownership of data, enabling research and innovation without compromising privacy or security. As Europe moves toward more digitized and well-connected health and research systems between Member States, creating data spaces under a common governance framework, is it time to think about data collaboration, rather than data sharing?

## Author contributions

AB drafted and edited the manuscript. RB, MH-A, AO, AJB, NH, WM, KE, CH, PV, DC, and DV-G provided their perspectives as members of the Neuronet WG and data sharing experts in IMI neurodegeneration projects, developing the manuscript content. LS and CD coordinated the Neuronet project, together with all authors. All authors provided critical comments on manuscript drafts and approved the final manuscript.

## Funding

This work was supported by funding from the Innovative Medicines Initiative 2 Joint Undertaking (JU) under grant agreement number 821513 (Neuronet). The IMI JU receives support from the EU’s Horizon 2020 research and innovation program and EFPIA, and the Parkinson’s Disease Society of the UK LBG.

## Conflict of interest

NH is an employee of Janssen Pharmaceutica NV and owns stock in Johnson & Johnson, but no product-related aspects. LS is an employee of Janssen Pharmaceutica NV. RB was employed by Aridhia Informatics Ltd.

The remaining authors declare that the research was conducted in the absence of any commercial or financial relationships that could be construed as a potential conflict of interest.

## Publisher’s note

All claims expressed in this article are solely those of the authors and do not necessarily represent those of their affiliated organizations, or those of the publisher, the editors and the reviewers. Any product that may be evaluated in this article, or claim that may be made by its manufacturer, is not guaranteed or endorsed by the publisher.

## Figures and Tables

**Table:** 

AD	Alzheimer’s disease
ADDI	AD Data Initiative
AMYPAD	Amyloid Imaging to Prevent Alzheimer’s Disease
CDM	Common data model
CMMI	Capability Maturity Model Integration^
CURATED	Check, Understand, Request, Augment, Transform, Evaluate, and Document
DPIA	Data Protection Impact Assessments
DUA	Data use agreements
EFPIA	European Federation of Pharmaceutical Industries and Association
EHDEN	European Health Data & Evidence Network
EHR	Electronic health records
EMIF	European Medical Information Framework
EPAD	European Prevention of Alzheimer’s Dementia
ETL	Extract, transform, load
EU	European Union
FAIR	Findable, accessible, interoperable, and reusable
GDPR	General Data Protection Regulation
IMI	Innovative Medicines Initiative
IP	Intellectual property
JU	Joint Undertaking
LCS	Longitudinal cohort study
ND	Neurodegenerative disorders
OHDSI	Observational Health Data Sciences & Informatics
OMOP	Observational Medical Outcomes Partnership
PC	Parent cohorts
PET	Positron emission tomography
PI	Principal investigators
PNHS	Prognostic and Natural History Study
PPP	Public-private partnerships
RMT	Remote measurement technologies
RWD	Real-world data
SHDN	Shared health data network
SME	Small and medium-sized enterprises
WG	Working group

## Footnotes

^1^ https://datacurationnetwork.org/
